# Public perception of mental health in Iraq

**DOI:** 10.1186/1752-4458-4-26

**Published:** 2010-10-11

**Authors:** Sabah Sadik, Marie Bradley, Saad Al-Hasoon, Rachel Jenkins

**Affiliations:** 1 Department of Psychiatry, International Medical Corps Iraq, Hai AlWahda, Mahla M904, Baghdad, Iraq; 2 Department of Adult Psychiatry, Leicestershire Mental Health Trust, Westcotes House, Westcotes Drive, Leicester, UK; 3 Department of Health Services and Population Research, Institute of Psychiatry, Kings College London, De Crespigny Park, London, UK

## Abstract

**Background:**

People who suffer from mental illness, the professionals who treat them, and indeed the actual concept of mental illness are all stigmatised in public perception and often receive very negative publicity. This paper looks at Iraq, which has a population of 30 million who are mainly Moslem. Mental health services and professionals have historically been sparse in Iraq with 1 psychiatrist per 300,000 before 2003 falling to 1 per million until recently and 1 primary care centre (40 Healthcare Workers including 4 General Practitioners) to 35,000 population, compared with 1 GP per 1700 population in the UK.

**Methods:**

We aimed to assess public attitudes and perceptions to mental illness. Participants were asked to complete a questionnaire (additional file 1), which was designed specifically for Iraqi contexts and was made available in 2 languages. The survey was carried out in 500 participants' homes across 2 districts of Baghdad.

**Results:**

The response rate of the survey was 86.4%. The paper shows respondents views on the aetiology of mental illness, perceptions of people with mental illness and attitudes towards care and treatment of people with mental illness.

**Conclusions:**

This survey of public attitudes towards mental illness in Iraq has shown that community opinion about the aetiology of mental illness is broadly compatible with scientific evidence, but understanding of the nature of mental illness, its implications for social participation and management remains negative in general.

## Background

Across the world, people with mental health problems, mental health services, mental health professionals and even the very concept of mental health receive negative publicity and are stigmatised in public perceptions [1,2], despite growing evidence of the importance of mental health for economic, social and human capital. Indeed the concept of mental capital for countries has recently been described [1]. Therefore increasing efforts are being made to challenge this negative publicity and stigma through anti-stigma campaigns, public education through schools, and the media etc [3].

Iraq is a Middle Eastern country of 30 Million largely Moslem population who have lived through extremely difficult conditions for many years, including physical privations, political repression and prolonged conflict. Mental health services in Iraq have historically been highly centralised in urban areas and hospital based, with 1 psychiatrist per 300,000 before 2003 falling to 1 per million until recently [4]. General primary health care services are relatively sparsely distributed, with 1 primary care centre (40 Healthcare Workers including 4 General Practitioners) to 35,000 population, compared with 1 GP per 1700 population in the UK.

The Iraq Ministry of Health strategy 2009 - 2011 has put primary care as the central plank of health care provision to the population, with emphases on competence, leadership, guidelines, standards and effective referral systems [5]. Mental health is one of the core priorities, along side maternal care, malnutrition, and non-communicable diseases.

Mental disorders are of particular concern in Iraq. A recent national survey found that the estimated lifetime prevalence of any disorder was 18.8% [6]. Cohort analysis documented significantly increasing lifetime prevalence of most disorders across generations. This was most pronounced for panic disorder and post-traumatic stress disorder, with lifetime-to-date prevalence 5.4-5.3 times as high at comparable ages in the youngest (ages 18-34) as oldest (ages 65+) cohorts. Anxiety disorders were the most common class of disorders (13.8%) and major depressive disorder (MDD) the most common disorder (7.2%). Twelve-month prevalence of any disorder was 13.6%, with 42.1% of cases classified mild, 36.0% moderate, and 21.9% serious. The survey also indicated that access to treatment is low (6.12%) [6].

In 2004 Al-Jawadi found that, 37.4% of children had mental health disorders (10.5% PTSD, 6% enuresis, and concluded the importance of mental health education [7].

The aim of the present study is to conduct a baseline survey of population attitudes towards mental illness in Iraq, at the start of a project which aimed to improve public perception of mental health in Iraq through a dual intervention which comprised education of primary care staff about mental health, and education of the public through a media campaign. The project was a collaboration between the Iraq Ministry of Health and the International Medical Corporation (a US based humanitarian NGO working in conflict areas).

## Methods

Administrative agreement for the study was obtained from the Iraq Ministry of Health, and ethics approval was obtained from the Ethics Committee of the National Council for Mental Health.

### Instruments

The questionnaire to assess public attitudes was developed in Iraq for the Iraqi context (see additional file 1) and included sections on socioeconomic data, previous contact with people with mental health problems, aetiology of mental illness, knowledge of people with mental illness and attitude towards people with mental health problems, and management of people with mental health problems. The questionnaire was administered to 30 IMC employees, and then following a discussion session to discuss the content and format of the questions, their comments were taken, and the questionnaire amended accordingly. There are no studies of its reliability. Answers were recorded on a questionnaire using a 5 point scale (agree, somewhat agree, neutral, somewhat disagree, disagree). The questionnaire was translated into Iraqi Arabic, and independently back translated by professional translators.

### Sample

The research design was a non- experimental random field research survey. The survey was conducted in Baghdad as travel across the rest of the country was difficult for logistic and security reasons. Five districts from Karkh and five districts from Rasafah were randomly selected to be demographically representative of the Baghdad population. A systematic random sampling procedure was used to select the sample units for the study with a randomly selected household as a starting point and a sampling interval of three. Thus the IMC interviewers interviewed all adults of both sexes present that day in every third house or apartment. Children under 18 were excluded.

The IMC interviewers had received a training session in the conduct of the interview by one of the authors (SA). Verbal consent was obtained from each participant, and the information was gathered anonymously. The IMC interviewer marked the questionnaire in accordance with the participant's responses.

### Sample size calculation

The maximum acceptable error for the estimation of proportions was set to 7% (0.07) and the design effect was assumed to be 2. The sample was further increased by 6% to account for contingencies such as non-response or data recording error.

**Figure 1:**
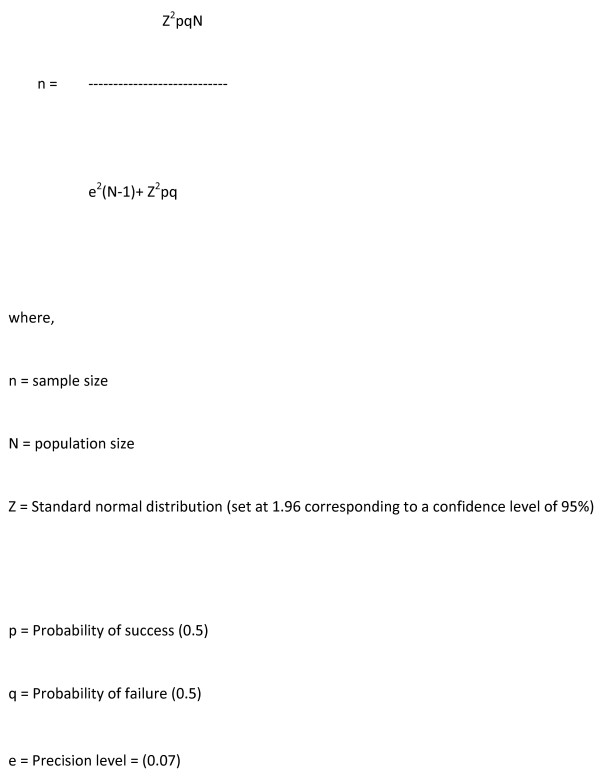
**Method of Sample size calculation**. where, n = sample size. N = population size. Z = Standard normal distribution (set at1.96 corresponding to a confidence level of 95%). p = Probability of success (0.5). q = Probability of failure (0.5). e = Precision level = (0.07)

Figure 1 shows the statistical formula was used to calculate the sample size for the study, and was calculated to be 380.

## Results

418 questionnaires out of 500 were returned giving a response rate of 86.4%.

Table 1 shows the socio-demographic breakdown of the sample. The gender distribution of the respondents was 225 male (55%) and 193 female (46%) resulting in a male-female ratio of 1.3:1. The age distribution was fairly even.

**Table 1:** Sociodemographic distribution of respondents

	Male%	(N = 232)	Female%	(N = 183)	Overall%	(N = 418)
***Age***						

< 20	9.91	23	10.38	19	10.29	42

21-30	28.45	66	30.05	55	29.19	122

31-40	32.33	75	33.33	61	32.54	136

41-50	15.95	37	17.49	32	16.51	70

51+	13.36	31	8.74	16	11.48	48

***Marital status***						

Single	27.83	64	24.86	45	26.81	112

Married	68.26	158	62.43	114	65.46	273

Divorced	1.30	4	3.87	7	2.42	11

Widowed	2.61	6	8.84	17	5.31	22

***Education***						

None	3.03	7	4.37	8	3.60	15

Elementary	14.29	34	18.03	33	15.83	66

Intermediate	19.48	45	20.77	38	20.14	84

Secondary	25.97	60	28.42	52	27.10	114

University or post-graduate	37.23	86	28.42	52	33.33	139

***Residence***						

Urban	95.20	220	97.77	178	96.35	402

Semi-urban	4.37	11	2.23	5	3.41	15

Rural	0.44	1	0	0	0.24	1

***Income***						

< 200,000 ID/month	16.67	38	49.01	89	29.60	123
200,000-400,000 ID/month	25.68	60	27.15	51	26.40	110

400,000-1,000,000 ID/month	44.14	102	19.87	36	34.40	144

> 1,000,000 ID/month	13.51	32	3.97	7	9.60	41

64% were married and 36% were either single, divorced or widowed, with marital status not recorded for 4 people. The vast majority lived in an urban environment with only 3.6% living in a rural environment within the two study districts. 39% of those interviewed either had no formal education or were educated up to intermediate level. 61% had attended both secondary and university level. People were less forthcoming about their income levels with 10% declining to answer.30% had an income of less than 200,000ID and 35% with an income of 400,000 to 1 million ID (approximately 1GBP = 1770 ID). 20% of respondents had had some prior contact with people with mental health problems. The sociodemographic distribution found in the Iraq Census is broadly similar [8,9].

Table 2 shows respondents' views on the aetiology of mental illness. It can be seen that around 60% of respondents agreed with the statement that mental illness is caused by brain disease. Half of respondents agreed with the statement that mental illness is caused by genetic inheritance. And nearly half agreed that substance abuse was the cause of mental illness. On the other hand, two thirds of respondents considered that mental illness was caused by something bad happening to you, while less than a third thought mental illness was God's punishment. Nearly two thirds viewed personal weakness as the cause of mental illness.

**Table 2:** Respondents' views on the aetiology of mental illness, by sex and age

		All	Male (N = 232)	Female (N = 183)	< 31 (N = 165)	31-50 (N = 205)	51+ (N = 48)
Mental Illness is caused by:							

Genetic inheritance	Agree	27.82	25.54	31.15	31.10	23.90	33.33

	Agree Somewhat	23.50	24.24	22.04	21.34	22.93	33.33

	Neutral	11.27	13.85	7.65	9.76	14.15	4.17

	Disagree somewhat	9.35	12.55	5.46	7.32	10.24	12.50

	Disagree	28.06	23.81	33.33	30.49	28.78	16.67

Substance Abuse	Agree	19.23	19.48	18.68	15.85	18.63	33.33

	Agree Somewhat	27.16	29.87	23.63	28.05	29.41	14.58

	Neutral	10.34	9.52	10.99	10.37	10.29	10.42

	Disagree somewhat	10.58	9.96	11.54	9.76	12.25	6.25

	Disagree	32.69	31.17	35.16	35.98	29.41	35.42

Bad Things happening to the person	Agree	42.48	40.61	45.56	45.96	39.22	44.68

	Agree Somewhat	24.76	24.89	25.00	27.33	23.53	21.28

	Neutral	17.48	19.65	13.89	10.56	23.04	17.02

	Disagree somewhat	7.52	7.42	7.22	6.83	7.84	8.51

	Disagree	7.77	7.42	8.33	9.32	6.37	8.51

Brain disease	Agree	35.59	37.12	33.15	32.92	34.31	50.00

	Agree Somewhat	25.91	29.69	21.55	27.95	25.98	18.75

	Neutral	8.96	10.04	7.18	11.18	6.86	10.42

	Disagree somewhat	6.30	6.11	6.63	6.21	7.84	0

	Disagree	23.24	17.03	31.49	21.74	25.00	20.83

Personal Weakness	Agree	38.13	39.83	36.61	36.59	37.56	45.83

	Agree Somewhat	21.10	23.38	18.58	26.83	16.59	20.83

	Neutral	14.63	14.72	14.21	10.98	18.05	12.50

	Disagree somewhat	10.07	8.66	11.48	8.54	11.71	8.33

	Disagree	16.07	13.42	19.13	17.07	16.10	12.50

God's Punishment	Agree	16.79	18.06	14.92	13.50	16.92	27.66

	Agree Somewhat	13.38	14.54	12.15	14.72	12.44	12.77

	Neutral	11.68	11.01	11.60	10.43	12.94	10.64

	Disagree somewhat	15.33	18.62	12.71	14.72	17.41	8.51

	Disagree	42.82	38.77	48.62	46.63	40.30	40.43

Tables 3 and 4 shows the respondents' perceptions and attitudes of people with mental illness. More than half of the respondents considered that people with mental illness are capable of work, and two thirds agreed that anyone can suffer from a mental illness. However, four fifths thought that people with mental health problems are largely to blame for their condition. Over half considered that people with mental illness are identifiable by their appearance, and just over half did not think that someone with a mental illness was capable of true friendships. Those surveyed were evenly split on whether someone with a mental illness was usually dangerous.

**Table 3:** Respondents' perceptions of people with mental illness

	Agree	Somewhat agree	Neutral	Somewhat disagree	Disagree
*Positive perception*					

Capable to work	25.73	28.64	13.35	16.26	16.02

Anybody can have mental illness	33.82	22.41	12.29	9.4	11.08

*Negative perception*					

Blame for own condition	61.93	21.45	8.67	3.61	4.34

Tell by physical appearance	25.67	33.74	9.78	11	19.8

Usually dangerous	16.71	26.88	12.35	21.79	22.28

Not capable of true friendship	33.66	20.34	16.22	17.68	12.11

**Table 4:** Attitude toward people with mental illness

	Agree	Somewhat agree	Neutral	Somewhat disagree	Disagree
*Positive perceptions*					

I could maintain friendship with someone with mental illness	34.47	23.54	13.59	12.38	16.02

I could marry someone with mental illness	8.56	11.25	9.54	20.54	50.12

Person with mental illness should have same rights	50.36	12.9	9	13.63	14.11

People generally caring and sympathetic towards people with Mental illness	39.28	14.94	11.57	17.83	16.39

*Negative perceptions*					

Mentally ill person should be prevented from having children	25.97	26.7	5.34	14.32	27.67

Mentally ill person should not get married	19.37	28.81	8.96	19.13	23.73

Mentally ill person should not be allowed to make decisions	23.47	25.43	11.25	22.49	17.36

One should avoid all contact with Mentally ill	21.12	23.06	15.29	17.23	23.3

I would be afraid to have conversation with Mentally Ill person	33.74	22.33	11.65	16.75	15.53

I would be upset and disturbed working on same job as mentally ill person	29.41	21.32	10.78	11.76	26.72

I would be ashamed if family member diagnosed with Mental illness	32.69	22.28	6.05	9.93	29.06

I would not want people to know if suffering from mental illness	52.9	22.46	4.59	8.21	11.84

Around half of respondents thought people with mental illness should not get married, and that people with mental disorders should not have children while just under half thought one should avoid all contact with people with mental illness. Just over half thought they could maintain a friendship with someone who had a mental illness, but less than one fifth thought they could marry someone with mental illness. Over half agreed that they would feel ashamed if a family member had a mental illness and over half would be afraid to have a conversation with a mentally ill person. While two thirds respondents thought that people with mental illness should have the same rights as anyone else, around half thought they would be disturbed about working in the same job as someone with a mental illness. Three quarters of respondents would not want people to know if they had a mental illness but just over half thought people were generally caring and sympathetic towards those with a mental illness.

Table 5 shows respondents' attitudes towards care and treatment of people with mental illness. Nearly half thought someone could recover from mental illness and nearly half of respondents disagreed with the statement that mental illness cannot be cured, but less than one fifth agreed that there were mental health services in their community. Two thirds of respondents thought that mental illness should not be hidden from their family. While nearly two thirds agreed with the statement that mentally ill people should be in an institution under supervision and control, just over two thirds also agreed that mental illness can be treated outside of a hospital. Only 15% considered that information about mental illness is available at their PHC, and only 14% thought that the PHC could provide good care for mental illnesses, but two thirds did consider they would feel comfortable discussing a mental health problem with someone at their PHC.

**Table 5:** respondents' attitudes towards care and treatment of people with mental illness.

	Agree	Somewhat agree	Neutral	Somewhat disagree	Disagree
*Positive perception*					

Mental illness can be treated outside a hospital	25.37	40	11.22	12.68	10.73

Majority of people with mental illnesses recover	19.56	28.12	8.07	23.47	20.78

I would feel comfortable discussing a mental health issue of family member or myself with someone at PHC	58.74	8.74	16.5	9.95	6.07

*Negative perception*					

One should hide mental illness from family	16.43	10.39	8.45	10.87	53.86

Mental illness cannot be cured	18.36	23.67	10.14	22.46	25.36

Mentally ill people should be in an institution to be under supervision and control	42.37	21.31	9.93	14.04	12.35

*Mental Health Service availability*					

Information about mental illness is available at my PHC	7.35	8.33	19.36	17.89	47.06

Mental health services available in my community	7.54	11.68	11.92	12.9	55.96

PHC clinics can provide good care for mental illnesses	8.29	6.34	13.17	11.95	60.24

As described above, the survey participants were asked about their willingness to form a range of personal relationships with people such as those described in the vignettes "marry someone with mental illness" (possible responses 'yes' or 'no') (see table 4). This information was then used to calculate a social distance score (see Table 6) where the minimum possible score was zero, indicating willingness to engage with the person in the vignette in all of the defined relationships, and the maximum score was five, indicating unwillingness to engage.

**Table 6:** Correlates of social distance scores

Variable	Mean Score	n	P value	Variable	Mean Score	n	P value
-Mentally ill person can't work				-Caring and sympathetic towards the person			

Agree		66	0.019	Agree		163	0.884
Agree		67		Agree		62	
somewhat		55		somewhat		48	
No response	1.68	118		No response	1.57	74	
Disagree		106		Disagree		68	
somewhat				somewhat			
Disagree				Disagree			

-Persons are usually dangerous				-Hiding self mental illness problems			

Agree		69	0.003	Agree		219	0.449
Agree		111		Agree		93	
somewhat		51		somewhat	1.04	19	
No response	2.06	90		No response		34	
Disagree		92		Disagree		49	
somewhat				somewhat			
Disagree				Disagree			

-Not capable of true friendship				-Mental illness can't cure			

Agree		139	0.826	Agree		76	0.294
Agree		84		Agree		98	
somewhat	1.54	67		somewhat		42	
No response		73		No response	2.13	93	
Disagree		50		Disagree	105		
somewhat				somewhat			
Disagree				Disagree			

-Prevent from having children				-Not allowed to decision making			

Agree		107	0.010	Agree		96	0.048
Agree		110		Agree		104	
somewhat		22		somewhat		46	
No response	1.91	59		No response	1.85	92	
Disagree		114		Disagree		71	
somewhatDisagree				somewhat			
Disagree				Disagree			

-Person should not get married				-Not maintain any friendship			

Agree	1.99	80	0.559	Agree	1.52	142	0.007
Agree		199		Agree		97	
somewhat		37		somewhat		56	
No response		79		No response		51	
Disagree		98		Disagree	1.52	66	
Somewhat				somewhat			
Disagree				Disagree			

-Avoid all contact mentally ill				-Marry someone with mentally ill			

Agree		87	0.189	Agree		35	0.002
Agree somewhat		95		Agree somewhat		46	
No response	1.99	63		No response	2.92	39	
Disagree		71		Disagree		84	
somewhat		96		somewhat		205	
Disagree				Disagree			

-Feel shame if family member is diagnosed				-Afraid to have conversion			

Agree		135	0.010	Agree		139	0.003
Agree		92		Agree		92	
somewhat		25		somewhat		48	
No response		41		No response		69	
Disagree	1.80	120		Disagree	1.58	64	
somewhat				somewhat			
Disagree				Disagree			

- Should have same rights like others				Cause by genetic inheritance			

Agree		207	0.367	Agree		116	0.207
Agree		53		Agree		98	
Somewhat		37		somewhat			
No response		56		No response		47	
Disagree	1.28	58		Disagree	1.86	39	
somewhat				somewhat		117	
Disagree				Disagree			

-Not work with mentally ill person				God's punishment			

Agree		120	0.876	Agree		69	0.766
Agree		87		Agree		55	
somewhat		44		somewhat		48	
No response	1.85	48		No response	2.54	63	
Disagree		109		Disagree		176	
somewhat				somewhat			
Disagree				Disagree			

-Hide mental illness problem of family				Cause by brain disease			

Agree		68	0.0001	Agree		147	0.152
Agree		43		Agree		107	
somewhat		35		somewhat		37	
No response	2.75	45		No response	1.56	26	
Disagree		223		Disagree		96	
somewhat				somewhat			
Disagree				Disagree			
*Causes of the problem*							
Cause by substance abuse				Cause by a personal weakness			

Agree		80	0.001	Agree		159	0.244
Agree		113		Agree		88	
somewhat		43		somewhat		61	
No response	2.10	44		No response	1.45	42	
Disagree		136		Disagree		67	
somewhat				somewhat			
Disagree				Disagree			

Cause by bad things happening							

Agree		175	0.001				
Agree		102					
somewhat		72					
No response	1.13	31					
Disagree		32					
somewhat							
Disagree							

The relationships between the social distance score and demographic, labelling and causation variables, perceived dangerousness, and previous contact were investigated. Greater social distance was significantly associated (P < 0.05) with: people with mental illness should not have children, being afraid to have a conversation with a mentally ill person, not maintaining friendship with a mentally ill person, considering that mentally ill people are usually dangerous, not wanting to marry someone with mental illness, hiding mental illness in the family, and being ashamed if people know that someone in the family is diagnosed with a mental illness. On the other hand reduced social distance was associated with considering that mentally ill people are capable of friendships, that people should be caring and sympathetic towards people with mental illness, that people would be upset or feel disturbed working in the same job with a mentally ill person, that mental illness cannot be cured, and that a mentally ill person should have the same rights as other people.

Sex, age, residence, marital status, income and previous contact with a family member or friend with a similar problem were not associated with increasing social distance scores; however education level was significantly associated with social distance (see Table 7)

**Table 7:** Multiple regression analysis to predict social distance using sociodemographic variables

Variable	B Coefficient	P value	95% Confidence Interval	
			
			Lower	Upper
Age	0.004	0.609	-.011	.019
Sex	-.016	0.920	-.337	.304
Marital Status	-.030	0.814	-.283	.223
Residence	-.183	0.593	-.854	.489
Education	-.132	0.047	-.269	.004
Income	-.071	0.450	-.257	.114
(Constant)	3.304		-.011	.019

• Dependent Variable: Would not marry someone with a mental illness

Those variables (namely educational level, and the following attitudes: Mentally ill persons prevent from having children, Feel shame if a person from the family is diagnosed, Hide mental illness problem from family, Not allow to take any decision even those concerning routine events, Not maintain a friendship with mentally ill person, Afraid to having conversion with mentally ill persons, Mentally ill persons are dangerous, Mentally ill person can't work) which are significantly associated (P < 0.05) with the social distance score were entered into a regression model (Table 8). This model showed that the final significant predictors of social distance were wanting to hide a mental illness problem from the family and not wanting to allow a person with mental illness to take their own decisions even those concerning routine events.

**Table 8:** Multiple regression analysis of predictors of social distance

Variable	B Coefficient	P value	95% Confidence Interval	
			
			Lower	Upper
Prevent from having children	0.036	0.440	-0.056	0.128
Feel shame if person from family diagnose	0.009	0.819	-0.071	0.089
Hide mental illness problem from family	0.151	0.001	0.069	0.234
Not allow to decision making	-0.234	0.001	-0.326	-0.141
Not maintain a friendship	0.039	0.474	-0.068	0.146
Afraid to having conversion	0.060	0.269	-0.047	0.166
Persons are dangerous	-0.082	0.086	-0.176	0.012
Person can't work	-0.037	0.441	-0.131	0.057
Education	-0.127	0.020	-0.234	-0.020
(Constant)	3.295		2.748	3.842

• Dependent Variable: Would not marry someone with a mental illness

## Discussion

The present study is the first systematic survey of attitudes towards people with mental illness in Iraq. Its design was constrained by the project's manpower, timeline, cost and security situation in Iraq, and thus the survey was conducted in Baghdad because of the logistic and security issues limiting travel, but the socio-demographic characteristics of our sample are representative of Baghdad and more broadly fairly representative of the urban Iraqi community, although our sample contained a higher proportion of university graduates than the population as a whole [10].

This baseline survey has shown that there is a high level of contact with people with mental health problems which may reflect a high prevalence of disorder, poor services or the community's acceptance of mentally ill people, or a combination of all three, and warrants further investigation. Attitudes towards mental illness in Iraq are very mixed, with large proportions of the population holding stigmatising attitudes towards people with mental illness in relation to treatment, work, marriage and recovery. The majority put the blame on the afflicted individual, avoided contact with them and would not openly discuss their own psychological problems.

On the other hand, the population did have a fairly reasonable understanding of the aetiology of mental illness, citing genetic factors, negative life events, brain disease and substance abuse as key causes although God's punishment and personal weakness were also viewed as major factors., Understanding of the nature of mental illness, its implications for social participation and management remains negative in general. However the majority accept patients' rights and the view that patients can be managed outside hospital, admit that the services at the PHC level are poor and would welcome developing such services. Social distance was associated with higher educational level, wanting to hide a mental illness problem from the family and not wanting to allow a person with mental illness to take their own decisions.

The limitations of our survey are that it only covered two districts, and did not include rural areas, and that the questionnaire was not previously tested for validity and reliability. We are not aware of a similar study in the Middle East with which to compare these results, but there are relevant studies in other regions of the world [11].

Most mental health literacy surveys have been largely conducted in western countries, with few studies in developing country contexts. Studies from western societies have shown that biological factors (diseases of the brain and genetic factors) and eventual factors (trauma and stress) are more likely to be considered causal [12-14], while in Africa, supernatural causes are widely considered [15-17], and a recent Nigerian survey found that urban dwelling, higher educational status, and familiarity with mental illness correlated with belief in biological and psychosocial causation, while rural dwelling correlated with belief in supernatural causes.

Adewuya et al 2008 [18], found that urbanicity, educational status, occupational status, age, and familiarity with mental illness are important independent correlates of multiple perceived causation of mental illness. A study in India of community beliefs about causes and risks for mental disorders, (Kermode et al 2009 [19], found that the most commonly acknowledged causes were a range of socio-economic factors, while neither supernatural causes nor biological explanation were widely endorsed.

As well as studies on mental health literacy, there have also been related studies about stigma about mental illness. As with mental health literacy, most research studies of stigma has been conducted in western countries but there are a small number in low and middle income countries [20-26]. Culture is likely to influence the experience, expression, and determinants of stigma and effectiveness of approaches to stigma reduction.

In India Kermode and colleagues [19], found that the main predictors of a variable of social distance from people with mental illness was perceiving the person as dangerous, while the main predictors of reduced social distance was being a volunteer health worker, and seeing the problem as a personal weakness. For depression, believing the cause to be family tension reduced social distance. For psychosis, labelling the illness as a mind/brain problem, a genetic problem or a lack of control over life increased social distance, and this may be due to the central importance of marriage in Indian culture. These findings suggest that promoting explanations around genetic and other physical causes may not always help stigma.

## Conclusions

Community opinion in Iraq about the aetiology of mental illness is broadly compatible with scientific evidence, However, understanding of the nature of mental illness, its implications for social participation and management remains negative. It is likely to be possible to build on the existing positive attitudes in the Iraqi population to enhance social inclusion of people with mental illness. There is therefore a need for well coordinated public education and for increased accessibility of effective mental health care through sustained primary care training, support and supervision about mental health.

## Competing interests

The authors declare that they have no competing interests.

## Authors' contributions

SS led the project and wrote first draft of the paper, MB contributed to the project delivery, SAH did the statistics, figures and tables. RJ led the later revisions of the paper. All authors read and approved the final manuscript.

## Supplementary Material

Additional file 1 **Public Perception of Mental Illness Questionnaire**. Click here for file
