# Supplementary material for: Public perception of mental health in Iraq
Source: Int J Ment Health Syst. 2010 Oct 11;4:26. doi: 10.1186/1752-4458-4-26 (PMC2964529; doi:10.1186/1752-4458-4-26)
Supplement: Additional file 1 — Public Perception of Mental Illness Questionnaire. [file 1752-4458-4-26-S1.DOC]

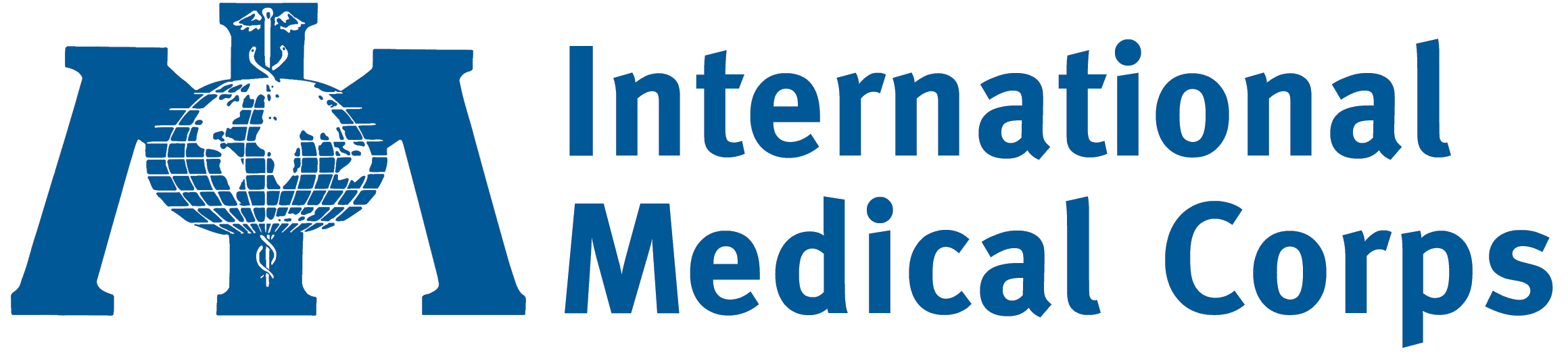

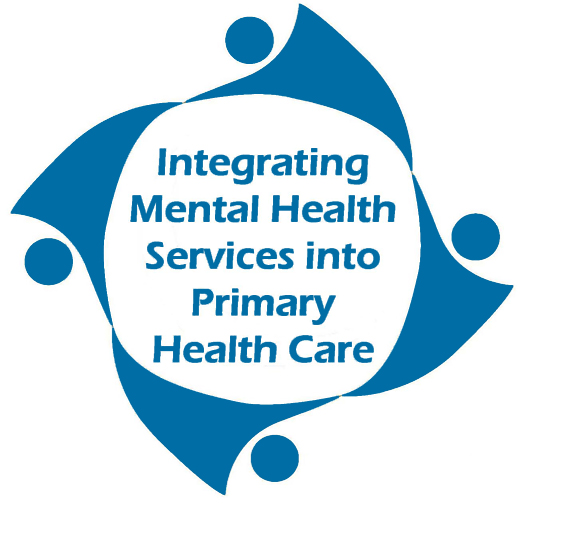
**Public Perceptions of Mental Illness in Iraq**

**Baseline Survey**

**الادراك الشعبي للامراض النفسيه في العراق**

**Date:** |__|__|/|__|__|/2009

**التاريخ Day Month**

**شهر يوم**

**Governorate: المحافظه**

**District: القطاع**

**PHC Catchment Area: الرعاية الصحية في المنطقة**

**DEMOGRAPHIC INFORMATION**

| **Age:** |__|__|العمر  **Sex:**  Male ذكر  الجنس  Femaleانثى | **Marital status:الحالة الزوجيه**   Singleاعزب   Marriedمتزوج   Divorcedمطلق   Widowedارمل | **Residence:السكن**   Urbanحضري   Semi-urbanشبه حضري   Ruralريفي |
| --- | --- | --- |

| **Education:التعليم**   Noneامي   Elementaryابتدائي   Intermediateمتوسطه   Secondaryاعدادي   University or higherجامعي /اعلى | **Income:الدخل**   <200,000 ID/monthاقل من 200 الف   200,000-400,000 ID/monthمابين 200 الف الى 400 الف   400,000-1,000,000 ID/monthما بين 400 الف الى المليون   >1,000,000 ID/monthاكثر من مليون |
| --- | --- |

**Would you describe yourself as having had significant previous contact with a person with mental illness?**

هل لديك شخص مصاب بمرض نفسي؟

 Yesنعم

 Noلا

| **CAUSE OF MENTAL ILLNESS**  **سبب المرض النفسي** | **موافق Agree** | **موافق احيانا**  **Agree Somewhat** | **عادي**  **Neutral** | **غير موافق احيانا** | **Disagree Somewhat** | **غير موافق Disagree** |
| --- | --- | --- | --- | --- | --- | --- |
| Mental illness is caused by genetic inheritance.  المرض النفسي سببه الجينات الوراثية | م A م ا AS ع N غ م ا DS غ م D | | | | | |
| Mental illness is caused by substance abuse.  المرض النفسي سببه المخدرات والكحول | م A م ا AS ع N غ م ا DS غ م D | | | | | |
| Mental illness is caused by bad things happening to you.  المرض النفسي سببه امر سيئ حدث لك | م A م اAS غ N غ م ا DS غ م D | | | | | |
| Mental illness is God's punishment.  المرض النفسي عقاب من رب العالمين | م A م ا AS غ N غ م ا DS غ م D | | | | | |
| Mental illness is caused by brain disease.  المرض النفسي سببه مرض في الدماغ | م A م ا AS غ N غ م ا DS غ م D | | | | | |
| Mental illness is caused by a personal weakness.  المرض النفسي سببه الضعف الذاتي او الشخصي | مA م ا AS غ N غ م ا DS غ م D | | | | | |
| **KNOWLEDGE OF PEOPLE WITH MENTAL ILLNESS**  **معلومات الناس عن الامراض النفسية** | **موافق Agree** | **موافق احيانا Agree Somewhat** | **عادي Neutral** | **غير موافق احيانا Disagree Somewhat** | | **غير موافق Disagree** |
| People with mental health problems are largely to blame for their own condition.  الناس الذين لديهم مشاكل في الصحة النفسيه يلومون الظروف | م A م ا AS ع N غ م ا DS غ م D | | | | | |
| One can always tell a mentally ill person by his or her physical appearance.  الانسان يستطيع ان يعرف المريض النفسي من خلال مظهره الجسمي | م A م ا AS ع N غ م ا DS غ م D | | | | | |
| Mentally ill persons are not capable of true friendships.  المرضى النفسيون لا يستطيعون عمل صداقات حقيقية | م A م ا AS ع N غ م ا DS غ م D | | | | | |
| Mentally ill persons can work.  المرضى النفسيون يستطيعون العمل | مA م ا AS ع N غ م ا DS غ م D | | | | | |
| Mentally ill persons are usually dangerous.  المرضى النفسيون خطرون عادة | م A م ا AS ع N غ م اDS غ م D | | | | | |
| Anyone can suffer from a mental illness.  كل شخص ممكن ان يعاني من المرض النفسي | م A م ا AS ع N غ م ا DS غ م D | | | | | |
|  | | | | | | |
| **ATTITUDE TOWARD PEOPLE WITH MENTAL ILLNESS**  **وجهة النظر حول المرض النفسي** | **موافق Agree** | **موافق احيانا Agree Somewhat** | **عادي Neutral** | **غير موافق احيانا Disagree Somewhat** | | **غير موافق Disagree** |
| The mentally ill should be prevented from having children.  المريض النفسي يجب ان يمنع عن الانجاب | م A م اAS ع N غ م ا DS غ م D | | | | | |
| The mentally ill should not get married.  المريض النفسي يجب ان لايتزوج | م A م ا AS عN غ م ا DS غ م D | | | | | |
| One should avoid all contact with the mentally ill.  الشخص يجب ان يتجنب التماس مع المريض النفسي | م A م ا AS عN غ م ا DS غ م D | | | | | |
| The mentally ill should not be allowed to make decisions, even those concerning routine events.  المريض النفسي يجب ان لا يسمح له بأتخاذ القرارات حتى المعتادة منها | م A م ا AS عN غ م ا DS غ م D | | | | | |
| I could maintain a friendship with someone with a mental illness.  استطيع ان ابقى بصداقة مع المريض النفسي | م A م اAS عN غ م ا DS غ م D | | | | | |
| I could marry someone with a mental illness.  استطيع ان اتزوج مريض نفسي | م A م ا AS ع N غ م ا DS غ م D | | | | | |
| I would be afraid to have a conversation with a mentally ill person.  قد أخاف عند تحدثي مع المريض النفسي | م A م ا AS ع N غ م ا DS غ م D | | | | | |
| People with mental health illnesses should have the same rights as anyone else.  الاشخاص الذين يعانون من الامراض النفسيه لديهم نفس حقوق الاصحاء | م A م ا AS ع N غ م ا DS غ م D | | | | | |
| I would be upset or disturbed about working on the same job as a mentally ill person.  انا اتهيج او انزعج عند قيامي بنفس العمل مع المريض النفسي | م A م ا AS عN غ م ا DS غ م D | | | | | |
| I would be ashamed if people knew that someone in my family had been diagnosed with a mental illness.  اشعر بخجل عندما يعرف الناس ان في عائلتي شخص مريض نفسي | م A م ا AS ع N غ م ا DS غ م D | | | | | |
| If I was suffering from a mental health illness, I wouldn't want people to know about it.  اذا كنت اعاني من مشاكل في الصحة النفسية لا ارغب بان يعرف احد | م A م ا AS ع N غ م ا DS غ م D | | | | | |
| People are generally caring and sympathetic towards people with mental illness.  الناس بصورة عامة يتعاطفون ويعتنون بالمرضى الذين يعانون من مشاكل بالصحة النفسية | م A م ا AS ع N غ م ا DS غ م D | | | | | |
| **CARE AND MANAGEMENT OF PEOPLE WITH MENTAL ILLNESS**  **الاعتناء والتعامل مع الاشخاص المريضون نفسيا** | **موافق Agree** | **موافق احيانا Agree Somewhat** | **عادي Neutral** | **غير موافق احيانا Disagree Somewhat** | | **غير موافق Disagree** |
| One should hide his/her mental illness from his/her family.  المريض النفسي يجب اخفاء مرضه عن عائلته | مA م ا AS ع N غ م ا DS غ م D | | | | | |
| There are mental health services available in my community.  خدمات الصحة النفسية متوفرة في مجتمعي | م A م ا AS غ N غ م ا DS غ م D | | | | | |
| Mental illness cannot be cured.المرض النفسي لا يشفى | م A م ا AS غ N غ م ا DS غ م D | | | | | |
| Mentally ill people should be in an institution where they are under supervision and control.  كل مريض نفسي يجب ان يكون في مصح حيث يكون تحت الاشراف والسيطرة | م A م ا AS ع N غ م اDS ع م D | | | | | |
| Mental illness can be treated outside a hospital.  المريض النفسي يمكن معالجته خارج المستشفى | مA م اAS ع N غ م ا DS غ م D | | | | | |
| Information about mental illness is available at my PHC.  المعلومات حول المرض النفسي متوفرة في المركز الصحي الأولي | مA م ا AS ع N غ م ا DS غ م D | | | | | |
| The majority of people with mental illnesses recover.  اغلبية المرضى النفسيون يشفون | م A م اAS ع N غ م ا DS غ م D | | | | | |
| Primary health care clinics can provide good care for mental illnesses.  مركز الرعاية الصحية الاولية يقدم رعاية جيدة للمرضى النفسيين | م A م ا AS عN غ م ا DS غ م D | | | | | |
| If I was concerned about a mental health issue with a member of my family or myself, I would feel comfortable discussing it with someone at my PHC.اذا كنت انا شخصيا مهتم بقضية الصحة النفسية او مع اي عضو من عائلتي سوف اشعر بالراحه في مناقشتها مع اي شخص اخر في الرعاية الصحية الاولية | مA AS ع N غ م ا DS غ م D | | | | | |
